# Liposomal amphotericin B prophylaxis in paediatrics: a systematic review

**DOI:** 10.1093/jac/dkaf171

**Published:** 2025-06-10

**Authors:** Emma V Thorley, Jennifer Hatch, Monica Li, Sharlene N Mashida, Elio Castagnola, Alessio Mesini, Thomas Lehrnbecher, Andreas H Groll, Adilia Warris, Laura Ferreras-Antolin

**Affiliations:** Centre for Neonatal and Paediatric Infection, City St. George’s, University of London, London, UK; Paediatric Infectious Diseases Unit, St George’s University Healthcare NHS Foundation Trust, London, UK; Pharmacy Department, St George’s University Healthcare NHS Foundation Trust, London, UK; Pharmacy Department, St George’s University Healthcare NHS Foundation Trust, London, UK; Paediatric Infectious Diseases Unit, St George’s University Healthcare NHS Foundation Trust, London, UK; Infectious Diseases Unit, IRCCS Istituto Giannina Gaslini, Genoa, Italy; Infectious Diseases Unit, IRCCS Istituto Giannina Gaslini, Genoa, Italy; Department of Pediatrics, Division of Pediatric Hematology, Oncology and Hemostaseology, Goethe University Frankfurt am Main, Frankfurt, Germany; Infectious Disease Research Program, Centre for Bone Marrow Transplantation and Department of Pediatric Hematology/Oncology, University Children’s Hospital, Münster, Germany; MRC Centre for Medical Mycology, Department of Biosciences, Faculty of Life and Health Sciences, University of Exeter, Exeter, UK; Centre for Neonatal and Paediatric Infection, City St. George’s, University of London, London, UK; Paediatric Infectious Diseases Unit, St George’s University Healthcare NHS Foundation Trust, London, UK; MRC Centre for Medical Mycology, Department of Biosciences, Faculty of Life and Health Sciences, University of Exeter, Exeter, UK

## Abstract

**Background:**

Liposomal amphotericin B (LAmB) is widely used for prophylaxis in paediatric patients at high risk of invasive fungal diseases (IFD) but its use is off-label and there is significant variability in dosage and frequency. This systematic review was conducted to evaluate the published data on prophylactic LAmB use in the paediatric population and to present the reported proportions of breakthrough IFD and the associated toxicity profile.

**Methods:**

EMBASE, Medline, Web of Science and the Cochrane Database were systematically searched for primary research reporting on the use of LAmB as prophylaxis for IFD in the paediatric population up to 7 December 2023, following Preferred Reporting Items for Systematic Reviews and Meta-Analyses (PRISMA) guidelines.

**Results:**

Twenty studies, comprising three clinical trials, 12 cohort studies, two point-prevalence surveys and three pharmacokinetic (PK) studies, with 2015 patients were included. A total of 717 cases presented individual patient data. Breakthrough IFD occurred in 7.2% (49/676). The most recognized side effects were hypokalaemia in 23.2% (125/538) and derangement of liver function tests in 15.0% (49/327). Discontinuation due to toxicity occurred in 6.0% (30/503) of patients. Of the four studies reporting PK data, two examined serum levels of LAmB, one analysed CSF levels and the remaining study peritoneal levels.

**Conclusions:**

Despite widespread use of prophylactic LAmB, this systematic review highlights the paucity of paediatric data supporting its use. The heterogeneity observed in populations, dosing regimens and study design prevents conclusions being reached on its efficacy or the superiority of one dosing regimen. Overall, there is a clear need for further high-quality robust clinical data and targeted PK studies.

## Introduction

Invasive fungal diseases (IFD) are a significant concern in the immunocompromised paediatric population.^1^ Primary antifungal prophylaxis should be considered for patients deemed at high risk (HR) of IFD [typically where natural risk of IFD is 10% or greater as per the European Conference on Infections in Leukaemia (ECIL) guidelines].^2^ The selection of an appropriate antifungal prophylaxis regimen depends on patient-specific criteria, including risk factors, age, pharmacokinetic properties, tolerability and potential side effects or interactions with concurrent medication, availability of paediatric formulations and the local fungal epidemiology.^3^ Liposomal amphotericin B (LAmB) is an intravenous polyene antifungal agent with a broad spectrum of antifungal activity *in vitro*. LAmB is not approved for prophylactic use by either the EMA in Europe, the FDA in the USA or the Medicines and Healthcare products Regulatory Agency in the UK.^4–6^ Despite the lack of approval, LAmB is frequently used as prophylaxis in different paediatric settings, albeit with significant variability in dosage and frequency.^7–9^ This systematic review aimed to describe the published evidence on the use of prophylactic LAmB in paediatric and young adult patients, with special interest in the occurrence of breakthrough infections and the agents toxicity profile.

## Methods

### Search strategy

We conducted a systematic literature review in line with Preferred Reporting Items for Systematic Reviews and Meta-Analyses (PRISMA) guidelines and registered the study on PROSPERO (International Prospective Register of Systematic Reviews: CRD42023414406).^10^ We searched EMBASE, Medline, Web of Science and the Cochrane Database for primary research reporting on the use of LAmB as prophylaxis for IFD in children and young adult patients (defined as those under 25 years). Oncology services in the UK are divided into paediatric and teenage and young adult, and therefore by including patients up to the age of 24 we aimed to ensure we captured the full paediatric population.^11^ The search was conducted over the last 35 years [original search 11 November 1987 to 11 November 2022, followed by an updated search 12 November 2022–07 December 2023; both searches are detailed in Table S1 (available as Supplementary data at *JAC* Online)]. LAmB was approved by the FDA in 1997 and thus this time period was chosen to ensure all trials relating to its use were included. The search was restricted to publications with a title and abstract in the English language; however, no publications where the main text was in an alternative language were identified. No country restrictions were used. Grey literature, including sources that were not published and/or peer reviewed, were not included in the search. A Boolean search strategy was developed containing terms related to LAmB, prophylaxis and children and/or young people (see Table S1 for full search terms).

Primary outcomes were: the description of the use of prophylactic LAmB in the paediatric and young adult population, the proportion of breakthrough infections and the reported safety and tolerance data. Where specified, pharmacokinetic (PK) data were included.

### Selection criteria

We included publications reporting primary data on the use of intravenous LAmB in paediatric and young adult patients from observational studies (cross-sectional, case-control or cohort studies, surveillance studies and case series including ≥3 patients) and trials, including randomized clinical trials (RCT) and non-randomized clinical trials with appropriate comparators.

Comments, editorials, literature and systematic reviews, letters, *in vitro* studies, case reports and case series with fewer than three patients were excluded. Publications were also excluded if they included only patients aged 25 years and over, or those with aggregated data where the outcomes of interest could not be extracted. Manuscripts reporting therapeutic use of LAmB were excluded.

Breakthrough infections were defined as any possible, probable or proven IFD while patients were on prophylactic LAmB.^12^ Unspecified IFD was used when none of these categories were reported. Toxicity and adverse effect data were collected and where used, the grade of toxicity was registered according to the CTCAE criteria.^13^

### Data screening, extraction and synthesis

Four reviewers (E.V.T., J.H., M.L. and S.N.M.) independently screened the titles and abstracts based on the inclusion and exclusion criteria for eligibility. The full-length articles were then retrieved for independent full review, data extraction and quality assessment (E.V.T., J.H., M.L. and S.N.M.). Any disagreements were resolved by frank discussion with the senior author (L.F.A.). Reasons for exclusions were recorded.

Where available, data was extracted on the following: study location and year, type of study, total number of patients under the age of 25 years included and the total number of patients given LAmB as prophylaxis. Due to the variety of study types included, differential reporting techniques were used including ‘patients’, ‘treatment episodes or courses of prophylaxis’ and ‘prescriptions’. Multiple separate episodes of prophylaxis over the study duration were also recorded (Table S2). Patient characteristics were detailed, including underlying conditions, as well as information on dosing, frequency of administration, duration and whether LAmB was primary or secondary prophylaxis. Where specified, primary outcome data was recorded, including the proportion of breakthrough IFD and rates of adverse events and drug discontinuations.

### Quality assessment

Certainty of evidence was assessed using the Grading of Recommendations, Assessment, Development and Evaluations (GRADE) framework, with a particular focus on the number of participants in each study and the risk of bias (Table S3).^14^ The risk of bias in each study was assessed using the GRADE approach detailed in Chapter 5 of the GRADE handbook [5.2.1: Study Limitations (Risk of Bias)], which includes key study limitations to consider.^14^

### Data analysis

Descriptive analysis was performed. Data on the usage of LAmB, rate of breakthrough IFD and associated adverse events were synthesized following the Synthesis Without Meta-Analysis guidelines.^15^ Studies were grouped according to the study design, observational studies (prospective and retrospective cohort studies, case-control studies and case series with three or more patients) versus clinical trials (randomized or quasi-randomized). Additionally, any reported PK data were analysed separately. Heterogeneity of reported effects was investigated by ordering tables referring to the study design, total number of participants/patients included and median age of included participants/patients.

## Results

### Overview of included studies

**Figure 1.**
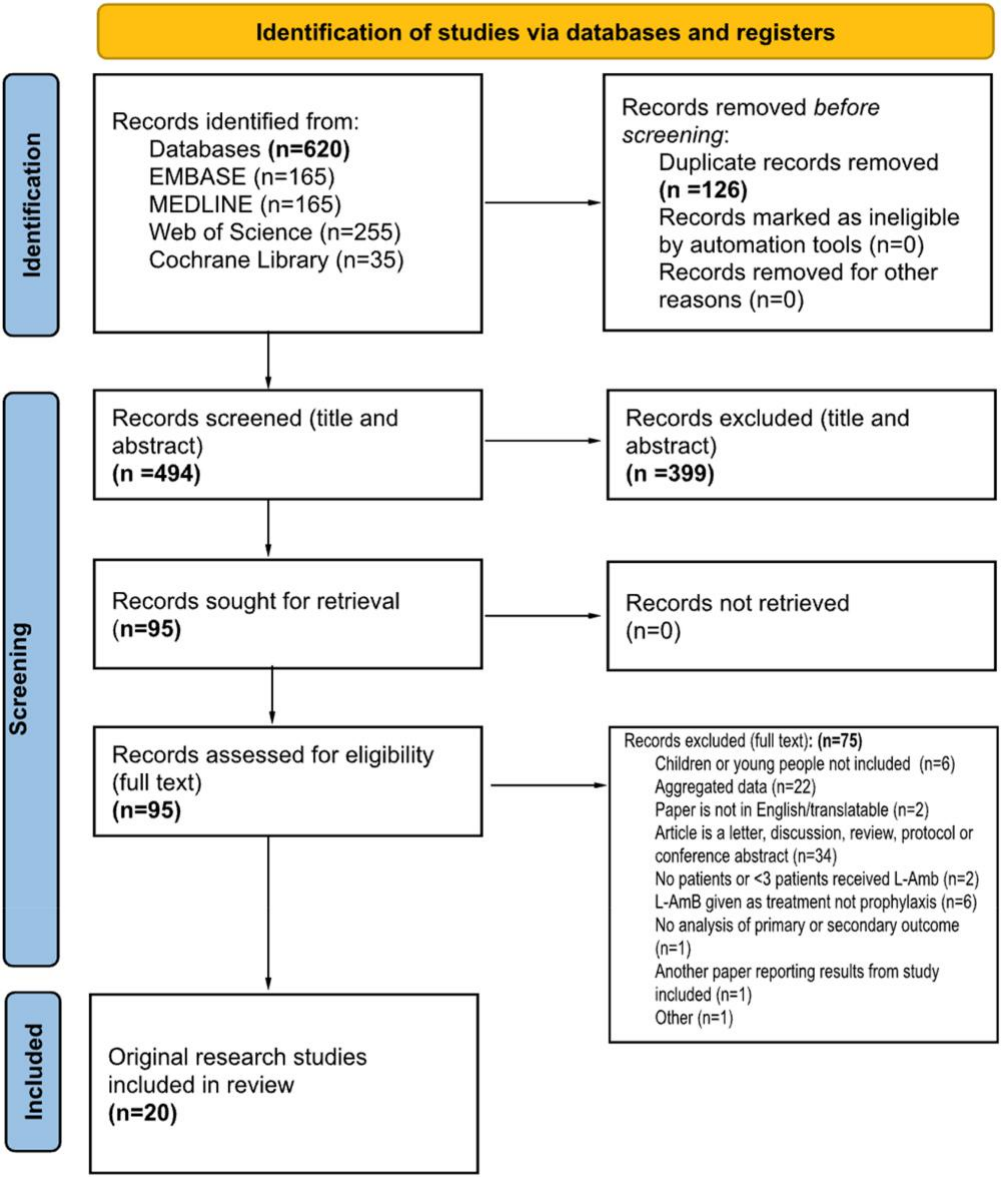
PRISMA flow diagram. PRISMA flow diagram of included data sources [including results from both the original search date (11 November 2022) and the search re-run (07 December 2023)].

We identified 494 articles. Twenty met the inclusion criteria for a full-length report, accounting for a total of 2015 patients (Figure 1). Adverse events, toxicities and/or the rate of breakthrough IFD were reported in 15 (75%) of the included studies. There were three clinical trials; two of them were randomized and one was placebo controlled (Table 1).

**Table 1.** Clinical trials included in the systematic review

Author and year of publication	Study type	Population	Intervention	Control	Outcome
Uhlenbrock *et al.* 2001^16^	Prospective, randomized clinical trial, non-blinded	29 high-risk paediatric haematology and oncology patients (including AML/HR-ALL/MDS etc)	Prophylaxis arm: thrice weekly 1 mg/kg LAmB	Early Intervention Arm: No prophylaxis	Incidence of IFD in prophylaxis arm 5/16 (31.3%, all probable) versus 6/13 in the early intervention arm (46.2%, 5 probable and 1 proved)
Roman *et al.* 2008^17^	Prospective, non-randomized clinical trial (pilot study)	51 paediatric patients undergoing 57 AlloSCT	Prophylactic LAmB 3 mg/kg/day from day 0–100	Historical cohort/comparative cohort not on trial	Incidence of IFD 5/51 (9.8%) and IMI 0/51 (0%) in LAmB prophylaxis cohort
Arrieta *et al.* 2010^18^	Prospective, randomized, placebo-control, open-label clinical trial (pilot study)	Very low birth weight premature infants	Prophylaxis with LAmB 5 mg/kg/week	Placebo (dextrose water)	Development of *Candida* colonization by 6 weeks postnatal age in 1/20 (5%) in LAmB prophylaxis group and 3/20 (15%) in placebo group; 0/20 (0%) subjects in LAmB group v versus 1/20 (5%) placebo subjects developed candidiasis

AML = acute myeloid leukaemia; HR-ALL = high-risk acute lymphoblastic leukaemia; MDS = myelodysplastic syndrome; IMI = invasive mould infection; AlloSCT = allogenic stem cell transplantation.

The studies reported data from nine different countries in North America and Europe, including Germany (*n* = 6), the USA (*n* = 5), Austria (*n* = 2), Spain (*n* = 2) the UK (*n* = 1), Denmark (*n* = 1), Poland (*n* = 1), Sweden (*n* = 1) and Italy (*n* = 1).^7, 8, 16–33^ The publication year spanned 25 years from 1997 to 2022.

All the manuscripts included children ≤18 years of age, with seven studies also including young adult patients (>18 and <25 years). The ages ranged from <7 days (age not specified) to 23 years of age.^16,18^ Table 2 summarizes other patient characteristics from the whole cohort of 2015 patients included. One single study presented data on the use of prophylactic LAmB in neonates exclusively.^18^

**Table 2.** Patient characteristics

Patient characteristics	Number of studies or patients included overall (total number of studies = 20; total number of patients = 2015)	
Age: by banding (reported by study)	Median age under 5 years	4/20 studies (20%)
	Median age between 5 and 10 years	12/20 studies (60%)
	Median age between 11 and 15 years	4/20 studies (20%)
Sex (recorded per patient, where reported)	Male	914/1608 patients (56.8%)
	Female	694/1608 patients (43.2%)
Underlying medical condition (recorded per patient, where reported)	Haematological malignancy ALL (inc. HR and relapsed) AML (inc. relapsed Non-Hodgkin’s lymphoma Hodgkin’s lymphoma Infantile leukaemia Burkitt’s lymphoma Other leukaemias Unspecified haematologial malignancy	742/2015 patients (36.8%) 423/742 (57.0%) 187/742 (25.2%) 32/742 (4.3%) 4/742 (0.5%) 4/742 (0.5%) 1/742 (0.1%) 26/742 (3.5%) 65/742 (8.8%)
	Post-HCT	473/2015 patients (23.5%)
	Post-liver or renal transplant	308/2015 patients (15.3%)
	Solid tumours	121/2015 patients (6.0%)
	Non-malignant haematological conditions	62/2015 patients (3.1%)
	VLBW premature neonates	40/2015 patients (2.0%)
	Other (including primary immune deficiency, congenital heart disease, PICU patients or unspecified)	269/2015 patients (13.3%)

VLBW = very low birthweight; PICU = paediatric intensive care unit; ALL = acute lymphoblastic leukaemia; AML = acute myeloid leukaemia. Characteristics presented from all patients in the 20 studies, including those on LAmB prophylaxis and those who were not.

### Use of prophylactic LAmB

Of the 20 studies included, 16 present data ‘per patient’ and account for a total of 1159 patients; of these 717 (61.9%) patients received prophylaxis with LAmB. Of the other four remaining studies, two were point-prevalence surveys where LAmB prophylaxis represented 32.7% (316/965) of all antifungal prophylactic prescriptions.^7,8^ The remaining two studies presented data as separate episodes of prophylaxis: one was a prospective observational study with 32 prophylactic LAmB episodes, and the other was a retrospective observational study describing 30 prophylactic LAmB episodes.^27,31^

There was significant variability in dosing regimens. The most common regimen described was 1 mg/kg daily, with 49.9% (337/676) patients reported in four studies.^24,26,29,32^ The different regimens are summarized in Table 3 with the respective IFD rates. In most studies LAmB was given as primary prophylaxis, except in the study by Allinson *et al.* in which 11 patients received secondary prophylaxis following a previous episode of probable or proven IFD.^32^ A detailed description of all studies is presented in Table S2.

**Table 3.** Cases of breakthrough IFD and IFD-associated mortality^a^

LAmB dosing	Study included	Number of patients who received prophylactic LAmB	IFD definition	IFD cases	Fungal infections detected (where specified)	IFD-specific mortality	Specific IFD (if reported)
1 mg/kg OD	Döring *et al.* 2012^29^	60	EORTC/MSGERC^12^	0/60		0/60 (0%)	N/A
	Mendoza-Palomar *et al.* 2020^24^	118 patients; 125 HCT episodes	EORTC/MSGERC^12^	10/118 (8.5%): all 10 with fungal species identified (proven)	2 *Candida* spp., 5 *Aspergillus* spp., *Trichosporon asahii*, *Fusarium solani*, *Rhizopus oryzae*	1/118 (0.8%)	Proven IFD with *Fusarium solani* (blood/skin/lung) and primary graft failure
	Teisseyre *et al.* 2007^26^	148	EORTC/MSGERC^12^	1/148 proven (0.7%)	*Aspergillus fumigatus*	0/148	N/A
	Allinson *et al.* 2008^32^	11	EORTC/MSGERC^12^	2/11 (18.2%)—1/11 possible 1/11 probable		2/11 (18.2%)	1 patient possible invasive pulmonary aspergillosis at day +135 post-HCT; 1 patient refractory graft failure and abundant *Aspergillus fumigatus* in BAL
	*Ringden et al.* 1997^b27^	30 (episodes of prophylaxis)	No guideline recorded	13/30 (43.3%)—all 13 with fungal species identified (proven)	*C. albicans* (8); *C. albicans* & *C. glabrata* (2); *C. albicans* & *C. parapsilosis* (2); *C. albicans* & *Saccaromyces cerevisiae* (1))	NR	N/A
1 mg/kg thrice weekly	Uhlenbrock *et al.* 2001^16^	16	No guideline recorded	5/16 probable (31.3%)		0/16	N/A
2.5 mg/kg twice weekly	Bochennek *et al.* 2011^30^	44 (46 HCT episodes)	EORTC/MSGERC^12^	1/44 (2.3%) possible		0/44 (0%)	N/A
	Vissing *et al.* 2021^25^	62	EORTC/MSGERC^12^	10/62 (16.1%)− 8/10 proven, 2/10 probable. 9 pulmonary infections, 1 cutaneous infections	7 *Aspergillus flavus* and 1 *Aspergillus fumigatus*	0/62 (0%)	N/A
3 mg/kg OD^c^	Roman *et al.* 2008^17^	51 patients; 57 HCT episodes	No guideline recorded	5/51 patients (9.8%) proven	0 IMI *Candida parasilosis* (*n* = 1); *Candida albicans* (*n* = 3); *Trichosporon beigelii* (*n* = 1).	0/51	N/A
	Satwani *et al.* 2009^20^	86	No guideline recorded	13/86 (15.1%)− all 13 with fungal species identified (proven)	RIC arm (*n* = 10): *Candida* spp. *n* = 7); *Aspergillus* spp. (*n* = 2); mucor (*n* = 1. MAC arm (*n* = 3): *Candida* spp. (*n* = 2); *Scedosporium* spp. (*n* = 1).	2/86 (2.3%)	1 pt with SAA and aGVHD developed *Candida lusitania*; 1 pt with Hodgkin Lymphoma and cGVHD with *Aspergillus fumigatus*
3 mg/kg thrice weekly	Meryk *et al.* 2020^22^	27	EORTC/MSGERC^12^	0/27		NR	N/A
5 mg/kg OW	Arrieta *et al.* 2010^18^	20	No guideline recorded	0/20 (0%) candidaemia; 1/20 (5%) secondary colonization.	1 patient in placebo arm developed candidaemia	0/20	N/A
10 mg/kg OW	Hand *et al.* 2014^19^	19	No guideline recorded	1/19 (5.3%) possible		1/19 (5.2%)	Possible/suspected fungal infection
	Mehta *et al*. 2006^21^	14	No guideline recorded	1/14 probable (7.1%)	Pulmonary nodule removed- pathology suggestive of fungal infection.	NR	N/A
Total	All studies including per patient data (excluding Ringdén)^16–22,24–26,29,30,32^	676	N/A	49/676 (7.2%)	37 proven (37/49, 75.5%); 9 probable (9/49, 18.4%); 3 possible (3/49, 6.1%)	6/635 (0.9%)	N/A

RIC = reduced intensity conditioning; MAC = myeloablative conditioning; OD = once daily; OW = once weekly; SAA = severe aplastic anaemia; GvHD = graft versus host disease; MSGERC = Mycoses Study Group Education and Research Consortium^12^

^a^Studies with no IFD data were not included.^7,8,23,28,31,33^

^b^The Ringdén trial is included in italics as the IFD rate is reported per prophylactic treatment episode rather than per patient.

^c^Although 3 mg/kg/day is typically considered a treatment dose, both authors clearly detail the use of 3 mg/kg/day IV from day 0 to day +100 post-HCT given as a prophylactic dose against IFD.

### Breakthrough invasive fungal disease (IFD)

Thirteen studies reported rates of breakthrough IFD per patient, which included 676 patients. (Table 3).^16–22,24–26,29,30,32^ The overall rate of IFD (proven, probable, possible) among patients given LAmB prophylaxis was 7.2% (49/676 patients), of which 75.5% (37/49) were proven, 18.4% (9/49) probable and 6.1% (3/49) possible. If the study by Arrieta *et al.* that only included neonates is excluded, the rate of IFD amongst the remaining population of immunosuppressed patients [including haematopoetic cell transplantation (HCT), haemato-oncology and solid organ transplant patients] is 7.5% (49/656).^18^ This ranged from no breakthrough cases in some studies to 30.3% in the RCT by Uhlenbrock *et al.*^16,18,22,29^ A further trial by Ringdén *et al.* reported 13 proven cases of IFD amongst 30 episodes of prophylaxis with LAmB (from a total of 78 episodes of prophylaxis in 61 patients), adding up to a total of 62 cases of breakthrough IFD, of which 50 were proven.^27^

The predominant pathogen among proven cases was *Candida* species (28/50, 56%) including one case of combined infection with *Candida* species and *Saccaromyces.*^17,18,20,24,27^ Proven invasive aspergillosis was diagnosed in 16 cases (16/50, 32%).^20,24–26^ There were two cases of proven invasive trichosporonosis and one case each of proven *Mucormycosis*, *Scedosporiosis*, *Fusarium solani* and *Rhizopus* species.^17,20,24^

Ten studies including 619 patients reported all-cause mortality, with a rate of 9.7% (60/619).^17–20,24–26,29,30,32^ Mortality attributable to IFD was assessed in these 10 studies plus one additional study and occurred in 0.9% (6/635) patients.^16–20,24–26,29,30,32^ Detailed data are documented in Table 3.

### Safety profile- toxicity and adverse events

Twelve of the studies detailed safety and tolerability data; however, in most cases, the degree of severity was not described. In a number of studies the adverse event rates reported were by HCT episode or by treatment episode rather than per patient.^17,24,27,30^ The most common adverse event reported was hypokalaemia (23.2%; 125/538, ranging from 4% to 80%) cases.^27,29^ Other adverse events described were altered liver function tests in 15.0% of the cases (49/327, ranging from 0% to 100%); renal impairment in 10.2% of the cases (55/537, ranging from 0% to 100%); infusion-related reactions in 11.4% of the cases (39/341, varying from 0% to 26.3%) and allergic reactions, including anaphylaxis in 4.4% of the patients (8/181, ranging from 0% to 14.8%) and hypomagnesaemia in 2.5% of cases (2/79).^18,19,22,27,29,32^ Table 4 details the data on toxicity and adverse events. The overall rate of LAmB discontinuation due to toxicity was reported in nine studies and was 6.0% (30/503) (see Table 4).^16,17,19,24,27,29–32^ The most common reason for discontinuation was infusion-related reactions in 40% (12/30), followed by nephrotoxicity in 30% (9/30), allergic reactions in 20% (6/30), isolated hepatotoxicity in 3.3% (1/30) and combined toxicities in 6.7% (2/30; one case of nephrotoxicity and hepatotoxicity, and one case of nephrotoxicity, hepatotoxicity and infusion-related reaction).

**Table 4.** Adverse events and toxicity reported with the use of prophylactic LAmB

Study included	LAmB dosing	Renal impairment	LFT derangement	Hypokaleamia	Hypomagnesaemia	Infusion-related reactions	Allergic reactions including anaphylaxis	LAmB discontinuation due to toxicity
Döring *et al.* 2012^29^	1 mg/kg OD	0/60 (0%)	15/60 (25%) AST >1.5; 13/60 (21.7%) ALT >1.5	48/60 (80%)	0/60 (0%)	5/60 (8.3%)	0/60 (0%)	4/60 (6.7%) (all infusion-related reactions)
Bochennek *et al.* 2011^30^	2.5 mg/kg twice weekly	7/184 (3.8%) episodes	Not reported	25/184 (13.6%) episodes	Not reported	4/44 (9.1%)	4/44 (9.1%)	4/44 (9.1%) (all allergic reactions; 3 at grade I/II and 1 grade III reaction)
Arrieta *et al.* 2010^18^	5 mg/kg once weekly	Not reported	0/20 (0%)	10/20 (50%)	Not reported	0/20 (0%)	0/20 (0%)	Not reported
Roman *et al.* 2008^17^	3 mg/kg OD	7/57 episodes (12.3%)	Raised AST 9/57 (15.8%); Raised bilirubin 10/57 (17.5%)	2/57 (3.5%)	Not reported	5/57 (8.8%)	Not reported	6/57 (10.5%) (all due to nephrotoxicity)
Uhlenbrock *et al*. 2001^16^	1 mg/kg thrice weekly	Not reported	Not reported	7/16 (43.8%)	Not reported	3/16 (18.8%)	Not reported	3/16 (18.8%) (all due to infusion-related reactions)
Meryk *et al.* 2020^22^	3 mg/kg thrice weekly	6/27 (22.2%)	Not reported	12/27 (44.4%)	Not reported	Not reported	4/27 (14.8%)	Not reported
Mendoza-Palomar *et al.*^a^, 2020^24^	1 mg/kg OD	11/125 (8.8%) episodes (all grade 1)	3/125 (2.4%) (all grade 1)	2/125 (1.6%)	Not reported	17/125 (13.6%)	Not reported	1/125 (0.8%) (due to multiple toxicities- nephrotoxicity/hepatotoxicity and infusion-related reaction)
Hand *et al*. 2014^19^	10 mg/kg once weekly	1/19 (5.3%)	Not reported	7/19 (36.8%) -no severe episodes	2/19 (10.5%)	5/19 (26.3%)	Not reported	5/19 (26.3%) (all infusion-related reactions)
Allinson *et al*. 2008^32^	1 mg/kg OD	11/11 (100%) deterioration in U&E (all resolved)	11/11 (100%) deterioration in LFTs	Not reported	Not reported	Not reported	Not reported	1/11 (9.1%) due to combined nephrotoxicity and hepatotoxicity
Stuecklin-Utsch *et al.* 2002^33^	1 mg/kg thrice weekly	1/24 (4.2%): Grade 1 Renal toxicity	1/24 (4.2%): Grade 1 hepatotoxicity	Not reported	Not reported	Not reported	Not reported	Not reported
Ringdén *et al.*^a^ 1997^27^	1 mg/kg OD	11/30 (36.7%)	Increased ALP in 9/30 (30%)	12/30 (40%)	Not reported	Not reported	0/30 (0%)	0/30 (0%)
Totals		55/537 (10.2%)	49/327 (15.0%)	125/538 (23.2%)	2/79 (2.5%)	39/341 (11.4%)	8/181 (4.4%)	24/362 (6.6%); including Kolve *et al.* study 30/503 (6.0%)

AST = aspartate aminotransferase; ALT = alanine aminotransferase; U&E = urea and electrolytes; LFT = liver function tests; ALP = alkaline phosphatase; OD = once daily, mg = milligram; kg = kilogram; OW = once weekly.

^a^Note that certain studies report the rate of adverse events by HCT episode^17,24,30^ or treatment episode^27^ as opposed to per patient.

### Pharmacokinetic studies and outcomes

There were four studies which presented PK data on the use of prophylactic LAmB (Table S4). Bochennek *et al.* analysed trough and peak serum amphotericin concentrations in a subset of five patients, following administration of 2.5 mg/kg twice weekly and after a median of 35 doses.^30^ The median trough level was 0.64 mg/L (0.22–6.19 mg/L) and the median peak level was 27.5 mg/L (24.4–56.2 mg/L). Mehta *et al.* reported results from 14 children, all younger than 10 years of age who were given 10 mg/kg once weekly.^21^ They described detectable amphotericin plasma levels on the seventh day before redosing and no accumulation after repeated doses. The mean amphotericin B concentration at 7 days was around the MICs for susceptible strains and the regimen was well tolerated, suggesting a potentially useful dosing strategy.^21^ Strenger *et al.* compared amphotericin B concentrations in serum versus CSF in 14 paediatric haemato-oncology patients, administered 3 mg/kg of LAmB on alternate days. The results supported previous animal studies with a low amphotericin B CSF concentration (1000-fold lower than serum) and a low transfer rate (0.13%, range 0.02%–0.92%).^23^ A final study by Tortora *et al*., examined amphotericin B concentrations in peritoneal fluid compared to plasma in six patients after receiving a one-off dose of 3 mg/kg.^28^ The peritoneal fluid *C*_max_ was significantly lower than plasma (*P* < 0.01) but both peritoneal *C*_max_ and *C*_min_ were in their established therapeutic range (0.2–3.0 mg/L).^28^

## Discussion

Our systematic review found limited literature on LAmB prophylaxis and demonstrated substantial heterogeneity in study design, dosing protocols and outcomes. The paediatric haemato-oncology and post-HCT patients were the most common recipients of LAmB prophylaxis, probably due to the potential contraindication of broad-spectrum azoles. Our analysis yielded an overall breakthrough infection rate of 7.2%, with significant disparities noted among prophylactic regimens. Hypokalaemia was the most frequently reported adverse effect, albeit with considerable incidence variability. The mean discontinuation rate due to toxicity was 6.0% in the analysed studies.

The most recent ECIL guidelines assign a level of evidence CII to the prophylactic use of LAmB.^2^ Similar to the adult population, optimal dosing, frequency and efficacy of LAmB as prophylaxis in paediatric patients is not well established.^34^ Our analysis underscores significant variability in prophylactic dosing strategies, ranging from daily administration to extended dosing regimens with varying dosages. Daily dosing at 1 mg/kg has been also reported as an empirical therapeutic option in patients with febrile neutropenia.^35^ These differences emphasize lack of well-powered studies aimed at defining the optimal prophylactic regimen based on pharmacokinetic and pharmacodynamic considerations. Similar uncertainties persist in the adult population, including the AmBiload study that trialled a higher dose of LAmB (10 mg/kg/day) that achieves maximal plasma LAmB levels but failed to show any improved clinical efficacy in a cohort of 201 adult patients with IFD.^34,36^ This further accentuates the need for well-designed studies to address these critical gaps in knowledge.

Two studies, Roman *et al.* and Satwani *et al.*, noted particularly high rates of IFD despite a prophylactic dose of LAmB as high as 3 mg/kg OD with a rate of 9.8% (5/51) and 15.1% (13/86) of proven IFD, respectively.^17,20^ This contrasts with the study from Bochennek *et al.* where in the study arm on LAmB at 2.5 mg/kg twice weekly no breakthrough infections were found.^30^ The overall rate of breakthrough IFD in patients on any LAmB prophylactic regimen was 7.2%, and of these 75.5% proven, 18.4% probable and 6.1% possible, but these results on efficacy are particularly difficult to interpret. First, the study design: there was significant heterogeneity among the studies, preventing meta-analysis of the study results. Moreover, there was a lack of robust RCT evidence in the paediatric and young adult population, particularly in comparing LAmB to alternative prophylactic antifungals or management strategies. Third, there was variability in dosing regimens implemented. Moreover, there was variability in the definition of IFD, with some studies not defining diagnosis or not following the EORTC (European Organisation for Research and Treatment of Cancer) criteria for disease classification.^12^ Finally, there was reporting bias with less data on uncomplicated cases on LAmB prophylaxis.

Nephrotoxicity, hepatotoxicity and infusion-related reactions are the most common adverse events described with LAmB.^34^ The overall toxicity rates in our review were lower than those described with LAmB given as treatment, with hypokalaemia and liver function test abnormalities occurring in 23.2% and 15.0% of patients, respectively, compared to approximately one-third of patients receiving LAmB as treatment in other reviews.^34,37^ Renal impairment, hypomagnesaemia and infusion-related reactions were less frequent but with significant variability among studies. We could not identify a more favourable toxicity profile in extended dosing regimens compared to daily administration. The overall drug discontinuation rate due to toxicity was 6.0%, which was lower compared to other studies where LAmB was given as treatment to similar mixed paediatric cohorts and where discontinuation rates were 2- to 3-fold higher.^38,39^ This might be explained in the context of higher dose or longer duration.^40,41^ No drug–drug interactions were reported in any of the included studies. LAmB has low rates of drug–drug interactions, which is particularly important in patients where broad-spectrum triazoles are contraindicated. This specifically applies to co-administration of vinca-alkaloids or small-molecule kinase inhibitors.^37,42^ Other drugs to consider for potential interactions are those that increase the risk of nephrotoxicity, including aminoglycosides, vancomycin, aciclovir and cyclophosphamide.^43^ However, evidence from RCTs in adult populations suggests that LAmB is generally well tolerated and an RCT with low dose LAmB versus placebo in a cohort of neutropenic adult patients with haematological malignancy found no grade 3 or 4 adverse events.^44–46^

Overall, there were limited PK data available on the use of prophylactic LAmB in the paediatric population, with only two studies examining serum amphotericin B concentrations after administration of LAmB.^21,30^ Bochennek *et al*. measured the concentration of total plasma amphotericin B, whereas Mehta *et al*. measured the concentration of non-lipid-complexed amphotericin in plasma.^21,30^ While the studies were not directly comparable, both present serum amphotericin B concentrations above the MICs for susceptible strains, although it should be noted that failure to completely disrupt the liposome results in an underestimation of the total concentration of amphotericin B within the matrix.^34^ The use of extended dosing regimens is based on how well amphotericin B distributes into the tissues and has a prolonged mean residence time in them at drug concentrations above the MIC for many fungi.^47^ However, meaningful PK/PD data in neonates and children is lacking and optimal dosing strategies are not known.^34^

This systematic review has several limitations. There was considerable heterogeneity between studies, and certain studies were not fully aligned with the primary outcome of the review. Additionally, as most studies were not RCTs, the results might be influenced by potential confounding factors, such as administration of other hepatotoxic or nephrotoxic agents or underlying conditions. The different use of ‘prescription episodes’ versus ‘patients’ or ‘courses of prophylaxis’ and the variability in prophylactic dosing regimens made comparisons between the studies challenging. A lack of consistency in the definitions of IFD, with not all studies using the EORTC/Mycoses Study Group Education and Research Consortium criteria, and different adverse events and toxicities grading should be noted.^12, 13^ Finally, although there were no reported cases of breakthrough IFD due to pathogens with known resistance to amphotericin B, the studies did not report information on *in vitro* antifungal susceptibility in these cases, which would have been of interest.

Although not approved for prophylaxis, LAmB may be a suitable alternative for specific populations, especially for haemato-oncology and post-bone marrow transplant patients, where other antifungal agents might cause clinically relevant drug–drug interactions. Extended dosing regimens are an attractive option due to cost reduction and the fact that they can be given in ambulatory settings, potentially enhancing patient’s quality of life. This systematic review highlights the paucity of paediatric clinical and pharmacokinetic/pharmacodynamic data supporting this prophylactic use. The evidence included shows the significant variability in patient populations, dosing regimens and study designs. This heterogeneity prevents the ability to reach strong conclusions on efficacy or the superiority of one prophylactic regimen above others, which needs to be urgently addressed. Large epidemiological studies through international collaboration and targeted pharmacokinetic and pharmacodynamic studies in paediatrics are required. Future work should also consider not only the comparison of LAmB with other existing antifungal alternatives but also with new compounds.^48^

## Supplementary Material

dkaf171_Supplementary_Data

## Transparency declarations

L.F.A. and A.W. are supported by the Medical Research Council Centre for Medical Mycology (MR/N006364/2) and the NIHR Exeter Biomedical Research Centre. A.W. has served as a consultant for Pfizer, Basilea and Gilead. E.T. is supported by an NIHR Academic Clinical Fellowship. A.H.G. has received grants from Gilead, Merck, Sharp & Dohme and Pfizer and has served as consultant to Amplyx, Astellas, Basilea, F2G, Gilead, Merck, Sharp & Dohme, Mundipharma, Pfizer and Scynexis. T.L. served in the speaker's bureau for Gilead Sciences, Merck/MSD, AstraZeneca, Pfizer, Sanofi Pasteur, and Recordati Pharma and as a consultant for Gilead Sciences, Merck/MSD, Pharming, PSI, Mundipharma, Roche, Recordati Pharma, Pfizer and received a research grant from Gilead Sciences. The views expressed are those of the author(s) and not necessarily those of the NIHR or the Department of Health and Social Care. All other authors have none to declare.

## Supplementary data

Tables S1 to S4 are available as Supplementary data at *JAC* Online.
