# Supplementary material for: Liposomal amphotericin B prophylaxis in paediatrics: a systematic review
Source: J Antimicrob Chemother. 2025 Jun 10;80(7):1792–802. doi: 10.1093/jac/dkaf171 (PMC12209837; doi:10.1093/jac/dkaf171)
Supplement: dkaf171_Supplementary_Data [file dkaf171_supplementary_data.docx]

**Supplementary Data**

**Table S1: Search Strategies** (Initial search date 11/11/22 (search strategy A); repeat search date 07/12/23 (search strategy B)

**S1.1A-** EMBASE via OVID (11/11/22)

| **Line** | **Search Term** | **Hits** |
| --- | --- | --- |
| 1 | Liposomal Amphotericin B | 3948 |
| 2 | Liposomal Amphotericin B/ | 12116 |
| 3 | Ambisome | 2976 |
| 6 | 1 OR 2 OR 3 | 13039 |
| 7 | Prevention/ | 288318 |
| 8 | Prophylaxis/ | 128721 |
| 9 | Chemoprophylaxis/ | 27278 |
| 10 | 7 OR 8 OR 9 | 428722 |
| 11 | Infant/ OR Child/ OR Paediatric/ OR Pediatric/ OR Adolescent/ | 3244290 |
| 12 | Young Adult | 511038 |
| 13 | 11 OR 12 | 3559993 |
| 14 | 6 AND 10 AND 13 | 166 |
| 15 | Limit to Humans | 160 |
| 16 | Limit to past 35 years | 160 |

**S1.1B-** EMBASE via OVID Re-Run (07/12/23)

| **Line** | **Search Term** | **Hits** |
| --- | --- | --- |
| 1 | Liposomal Amphotericin B | 4284 |
| 2 | Liposomal Amphotericin B/ | 12974 |
| 3 | Ambisome | 3056 |
| 6 | 1 OR 2 OR 3 | 13944 |
| 7 | Prevention/ | 303775 |
| 8 | Prophylaxis/ | 138922 |
| 9 | Chemoprophylaxis/ | 28477 |
| 10 | 7 OR 8 OR 9 | 453900 |
| 11 | Infant/ OR Child/ OR Paediatric/ OR Pediatric/ OR Adolescent/ | 3451616 |
| 12 | Young Adult | 560819 |
| 13 | 11 OR 12 | 3801877 |
| 14 | 6 AND 10 AND 13 | 178 |
| 15 | Limit to Humans | 172 |
| 16 | Limit to 12/11/2022- 07/12/2023 | 5 |

**S1.2A -** Medline via OVID (11/11/22)

| **Line** | **Search Term** | **Hits** |
| --- | --- | --- |
| 1 | Liposomal Amphotericin B.mp | 3615 |
| 2 | Ambisome.mp | 600 |
| 5 | 1 OR 2 | 3795 |
| 6 | Prevention | 1869437 |
| 7 | Prophylaxis | 121504 |
| 8 | Chemoprophylaxis | 6558 |
| 9 | 6 OR 7 OR 8 | 1923206 |
| 10 | Infant* OR Child* OR Paediatric* OR Pediatric* OR Adolescent* | 4487488 |
| 11 | “Young Adult*” | 1071032 |
| 12 | 10 OR 11 | 4949628 |
| 13 | 5 AND 9 AND 12 | 173 |
| 14 | Limit to Humans | 162 |
| 15 | Limit to past 35 years | 162 |

**S1.2B -** Medline via OVID Re-Run (07/12/23)

| **Line** | **Search Term** | **Hits** |
| --- | --- | --- |
| 1 | Liposomal Amphotericin B.mp | 3808 |
| 2 | Ambisome.mp | 621 |
| 5 | 1 OR 2 | 3997 |
| 6 | Prevention | 1955627 |
| 7 | Prophylaxis | 127929 |
| 8 | Chemoprophylaxis | 6776 |
| 9 | 6 OR 7 OR 8 | 2012571 |
| 10 | Infant* OR Child* OR Paediatric* OR Pediatric* OR Adolescent* | 4646914 |
| 11 | “Young Adult*” | 1094320 |
| 12 | 10 OR 11 | 5119424 |
| 13 | 5 AND 9 AND 12 | 177 |
| 14 | Limit to Humans | 165 |
| 15 | Limit to 12/11/2022-07/12/2023 | 3 |

**S1.3A**- Web of Science (11/11/22)

| **Line** | **Search Term** | **Hits** |
| --- | --- | --- |
| 1 | Liposomal Amphotericin B | 5135 |
| 2 | Ambisome | 1072 |
| 3 | 1 OR 2 | 5497 |
| 4 | Prevention | 1035612 |
| 5 | Prophylaxis | 126225 |
| 6 | Chemoprophylaxis | 6149 |
| 7 | 4 OR 5 OR 6 | 1129510 |
| 8 | Infant* OR Child* or Paediatric* OR Pediatric* OR Adolescent* | 4199342 |
| 9 | Young Adult | 307379 |
| 10 | 8 OR 9 | 3006495 |
| 11 | 3 AND 7 AND 10 | 246 |
| 12 | Limit to Humans | 246 |
| 13 | Limit to past 35 years | 246 |

**S1.3B-** Web of Science Re-Run (07/12/23)

| **Line** | **Search Term** | **Hits** |
| --- | --- | --- |
| 1 | Liposomal Amphotericin B | 5416 |
| 2 | Ambisome | 1092 |
| 3 | 1 OR 2 | 5788 |
| 4 | Prevention | 1115450 |
| 5 | Prophylaxis | 133577 |
| 6 | Chemoprophylaxis | 6383 |
| 7 | 4 OR 5 OR 6 | 1214288 |
| 8 | Infant* OR Child* or Paediatric* OR Pediatric* OR Adolescent* | 4224656 |
| 9 | Young Adult | 330953 |
| 10 | 8 OR 9 | 4415104 |
| 11 | 3 AND 7 AND 10 | 256 |
| 12 | Limit to Humans | 256 |
| 13 | Limit to 2022-11-12 to 2023-12-07 | 9 |

**S1.4A -** Cochrane Library (11/11/22)

| **Line** | **Search Term** | **Hits** |
| --- | --- | --- |
| 1 | Liposomal Amphotericin B OR Ambisome | 395 trials; 7 cochrane reviews; 1 editorial; 0 clinical answers |
| 2 | Prevention OR Prophylaxis OR Chemoprophylaxis | 264493 trials; 3504 cochrane reviews; 363 cochrane protocols; 53 editorials; 10 special collections; 418 clinical answers |
| 3 | Paediatric* OR Pediatric* OR infant* OR child* OR adolescent* OR “Young Adult” | 356462 trials; 3223 cochrane reviews; 290 cochrane protocols; 43 editorials; 11 special collections; 448 clinical answers |
| 4 | 1 AND 2 AND 3 | 34 trials; 0 cochrane reviews; 1 editorial |
| 5 | Limited to last 35 years | 34 trials; 0 cochrane reviews; 1 editorial |

**S1.4B -** Cochrane Library Re-Run (07/12/23)

| **Line** | **Search Term** | **Hits** |
| --- | --- | --- |
| 1 | Liposomal Amphotericin B OR Ambisome | 413 trials; 18 cochrane reviews; 2 cochrane protocols; 1 editorial; 2 clinical answers |
| 2 | Prevention OR Prophylaxis OR Chemoprophylaxis | 234297 trials; 4448 cochrane reviews; 597 cochrane protocols; 42 editorials; 15 special collections; 416 clinical answers |
| 3 | Paediatric* OR Pediatric* OR infant* OR child* OR adolescent* OR “Young Adult” | 405549 trials; 5605 cochrane reviews; 916 cochrane protocols; 73 editorials; 14 special collections; 1158 clinical answers |
| 4 | 1 AND 2 AND 3 | 39 trials; 0 cochrane reviews; 1 editorial |
| 5 | Limited to November 2022- December 2023 | 1 trial; 0 cochrane reviews; 0 editorial |

**Table S2: Table with all Included Studies**

| **Study ID** | **Author and Year of Publication** | **Country** | **Study Type** | **Population** | **Age of Patients** | **Sex** | **Number of Prescription Episodes of/ patients who received prophylactic LAmB/ patients** | **Dosing and Frequency of LAmB** | **Primary Outcome** | **Key Results** |
| --- | --- | --- | --- | --- | --- | --- | --- | --- | --- | --- |
| 1 | Ferreras-Antolin *et al.* 2022^7^ | UK | Observational prospective point prevalence study | 656 paediatric patients including haematological, oncological malignancy patients and transplant. | All 90 days-18 years. Median 6.4 (IQR 2.5-11.3) | 363 M: 293 F | 275/1258 (21.9%) total prescriptions (275/890 (30.9%) prophylactic prescriptions) | NR | Point prevalence study on antifungal prescriptions in the UK | Large PPS in UK showing high frequency of prophylactic LAmB prescriptions, with almost LAmB constituting almost a third (30.9%) of total prophylaxis prescriptions. |
| 2 | Mendoza-Palomar *et al.* 2021^8^ | Spain | Observational prospective point prevalence study | 55 paediatric patients including malignancy and HCT. | All <18 years. Median 8.7 years (IQR 2.4-13.8) | 29 M: 26 F | 41/119 (34.5%) total prescriptions (41/75 (54.7%) prophylactic prescriptions) | NR | Point prevalence study on antifungal prescriptions in Spain | PPS showing that LAmB represents over half of prescriptions for prophylactic antifungals (54.7%). |
| 3 | Vissing *et al.* 2021 ^25^ | Denmark | Retrospective observational study | 62 children with high-risk leukaemia | All <18 years. | NR | **62** | 2.5mg/kg twice weekly | Efficacy of prophylactic LAmB in preventing invasive aspergillosis | Breakthrough IFD rate 16.1% (10/62). 8 proven cases of invasive aspergillosis (7 aspergillus flavus and 1 aspergillus fumigatus) and 2 probable cases. |
| 4 | Döring *et al.* 2012 ^29^ | Germany | Retrospective observational study | 120 paediatric allogenic HCT patients | All <18 years. Median age 7.5 years. | 33 M: 27 F | **60** | 1mg/kg OD (escalated to 3mg/kg if suspicion of fungal infection) | Comparison in efficacy between prophylactic LAmB and caspofungin in preventing IFD | No cases of breakthrough IFD in LAmB arm. One case of probable invasive aspergillosis in caspofungin arm. |
| 5 | Bochennek *et al.* 2011 ^30^ | Germany | Prospective observational study & PK study | 83 high risk haematology patients (*81 malignant and 2 non-malignant); 388 individual dose episodes antifungal prophylaxis* | Age 6 months- 21 years. Median age 7.7 years. | Study group- 24 M: 20 F | **46** cases in 44 patients; (187 dose episodes) | 2.5mg/kg twice weekly | Efficacy of prophylactic LAmB in preventing IFD and PK data on LAmB serum concentrations | Breakthrough IFD rate 2.3% (1/44)- 1 possible case.  PK: Median Cmax 27.5mg/L and median Cmin 0.64mg/L after median 35 doses of LAmB. |
| 6 | Arrieta *et al.* 2010 ^18^ | USA | Prospective, randomised, placebo-controlled clinical trial (pilot study) | 40 Very Low Birth Weight premature neonates | All < 7 days old, <32 weeks gestation | 24 M: 16 F | **20** | 5mg/kg once weekly | Efficacy of prophylactic LAmB in preventing secondary colonisation with candida | No cases of breakthrough IFD in the LAmB treatment arm; 1 patient in the placebo arm developed candidaemia (5%, 1/20).  Hypokalaemia reported in 50% of LAmB patients (10/20). |
| 7 | Roman *et al.* 2008 ^17^ | USA | Prospective, non-randomised clinical trial | 51 allogenic HCT patients (57 episodes of HCT) | Age 6 months- 21 years. Median age 6 years. | 32 M:19 F | 51 patients; **57** HCT episodes | 3mg/kg OD | Efficacy and safety of prophylactic LAmB in preventing IMI in the first 100 days post AlloSCT | Breakthrough IFD rate 9.8% (5/51). Five proven infections, 4 with candida and 1 trichosporon. Renal impairment in 12.3% (7/57), requiring discontinuation of LAmB in the majority (85.7%, 6/7). |
| 8 | Uhlenbrock *et al.* 2001 ^16^ | Germany | Prospective, randomised clinical trial | 29 high risk haematology patients (17 malignant; 2 non-malignant; 10 HCT) | Age 0-23 years. Median age 9 years. | 20 M: 9 F | **16** | 1mg/kg thrice weekly | The incidence of IFD following prophylactic LAmB versus early intervention with LAmB | Breakthrough IFD rate 31.3% (5/16), with all cases deemed probable. Hypokalaemia noted in 43.8% (7/16) with 18.8% (3/16) suffering infusion-related reactions necessitating cessation of LAmB. |
| 9 | Meryk *et al.* 2020 ^22^ | Austria | Retrospective observational study | 198 haematological malignancy patients | Age 2.74- 6.84 years. Median age 4.69 years. | 109 M: 89 F | **27** (*high-risk patients in 2010 onwards cohort)* | 3-5mg/kg thrice weekly | Tolerability of prophylactic LAmB and efficacy in preventing IFD | No reported cases of breakthrough IFD. Hypokalaemia reported in 44.4% (12/27), renal impairment in 22.2% (6/27) and allergic reactions in 14.8% (4/27). |
| 10 | Mendoza-Palomar *et al.* 2020 ^24^ | Spain | Retrospective observational study | 118 allogenic HCT patients (125 HCT procedures) | All <18 years. Median age 7.2 years (IQR 4.2-11.5 years) | 73 M: 45 F | **118** | 1mg/kg OD | Efficacy and safety of LAmB as primary antifungal prophylaxis | Breakthrough IFD rate 8.5% (10/118), with 1 case of IFD-specific mortality secondary to proven IFD with fusarium solani. 13.6% (17/125) suffered infusion-related reactions, but only 1 patient discontinued LAmB due to toxicity. |
| 11 | Hand *et al.* 2014 ^19^ | USA | Retrospective observational study | 19 paediatric cancer patients *(16 ALL patients; 3 oncology patients)* | Age 2 - 18 years. Mean age 6.5 years. | 12 M: 7 F | **19** *(total 96 prophylactic LAmB infusion doses)* | 10mg/kg once weekly | Safety and tolerability of prophylactic LAmB | Breakthrough IFD rate 5.3% (1/19)- 1 possible case. Hypokalaemia reported in 36.8% (7/19) and hypomagnaesaemia in 10.5% (2/19). Infusion-related reactions occurred in 26.3% (5/19)- all requiring treatment discontinuation. |
| 12 | Strenger *et al.* 2014 ^23^ | Austria | PK study | 14 paediatric patients: *11 haematological malignancy and 3 solid tumour patients* | Age 1.4-19.5 years. Median age 7.6 years. | 11 M: 3 F | **14** | 3mg/kg alternate days | Assessment of the pharmacokinetics of LAmB transfer to CSF | Median transfer rate of 0.13% from serum to CSF. Clear correlation between time after drug infusion and transfer rate.  CSF levels maintained a steady state for >48 hours. |
| 13 | Satwani *et al.* 2009 ^20^ | USA | Retrospective observational study | 86 allogenic HCT patients | All <18 years. Median age 7.5 years. | 52 M: 34 F | **86** | 3mg/kg OD | Incidence of IFD whilst on prophylactic LAmB on Reduced-Intensity (RIC) versus Myeloablative Conditioning (MAC) | Overall breakthrough IFD in 15.1% (13/86). Ten cases in RIC arm (7 with candida, 2 aspergillus and 1 mucor) and 3 cases in MAC arm (2 candida cases, 1 case of scedosporium). Two deaths related to IFD in patients with GvHD. |
| 14 | Kolve *et al.* 2009 ^31^ | Germany | Prospective observational study | 84 paediatric patients: *55 haematological malignancy; 17 solid tumour; 12 other (141 courses of LAmB)* | Age 0.2-20 years. Median age 11 years. | 46 M: 38 F | 32/141 courses given as prophylaxis (22.7%) | *Median daily dose 2.8mg/kg- not separated treatment vs prophylaxis* | Safety, Tolerability and Efficacy of prophylactic (and empirical) LAmB | Breakthrough IFD rate is presented in aggregated data with empirical therapy, however 96.9% (31/32) courses of prophylaxis were completed successfully (without discontinuation due to toxicity, breakthrough infection or mortality). |
| 15 | Allinson *et al.* 2008 ^32^ | Germany | Prospective observational study | 11 HCT patients with acute leukaemia; secondary prophylaxis | Age 11-18 years. Median age 14 years. | 4 M: 7 F | **11** | 1mg/kg OD | Efficacy of secondary prophylaxis with LAmB in patients with prior presumed or proven invasive pulmonary aspergillosis | Breakthrough IFD rate of 18.2% (2/11) with 1 possible and 1 probable case of invasive pulmonary aspergillosis. Both patients died, thought to be secondary to their IFD.  All patients suffered renal impairment and derangement of LFTs, although only 1 patient discontinued LAmB due to toxicity. |
| 16 | Teisseyre *et al.* 2007 ^26^ | Poland | Retrospective observational study | 277 patients post liver transplant | Age 1.1-20 years. Median age 7.5 years. | NR | **148** | 1mg/kg OD | Incidence of aspergillosis in liver transplant patients given prophylactic LAmB | Breakthrough IFD rate of 0.7% (1/148)- 1 proven case of aspergillus fumigatus. |
| 17 | Mehta *et al.* 2006 ^21^ | USA | PK study | 14 HCT patients | Age 4.5 months -9 years 9 months. Median age 3.1 years. | 9 M: 5 F | **14** | 10mg/kg once weekly | Assessing pharmacokinetics of prophylactic LAmB and attainment of therapeutic concentrations throughout dosing interval | No significant difference found between the Cmax after first dose versus 4^th^ dose. AUC significantly higher after 4^th^ dose versus first dose (p<0.05). Median elimination half life 45 hours. |
| 18 | Stuecklin-Utsch *et al.* 2002 ^33^ | Germany | Retrospective observational study | 31 paediatric patients with haematologicalmalignancy (12) or solid tumours (17) or non-malignant (2) | Age 9 months-17 years. Median age 4 years. | 17 M:14 F | **24** | 1mg/kg thrice weekly | Assessment of pancreatic toxicity following prophylactic LAmB | A transient increase in serum lipase was noted in 16.1% (5/31) patients, with clinical pancreatitis diagnosed in 3 of these patients (9.7% of the overall cohort). |
| 19 | Ringdén *et al.* 1997 ^27^ | Sweden | Retrospective observational study | 61 paediatric patients- 36 BMT, 25 liver or renal transplant (78 courses total) | Age 1-16 years. Median age 6 years. | 36 M: 25 F | 30/78 episodes (38.5%) | Median 1mg/kg/day | Tolerability, safety and efficacy of prophylactic (and therapeutic) LAmB | Breakthrough IFD rate 43.3% (13/30), all with proven candida species. 36.7% (11/30) suffered renal impairment, 80% (9/30) LFT derangement and 40% (12/30) hypokalaemia. |
| 20 | Tortora *et al.* 2022 ^28^ | Italy | PK study | 6 paediatric liver transplant patients | Age 5 months- 20 years. Median age 13.6 years. | NR | **3** | 3mg/kg (as a single dose) | Evaluation of peritoneal levels of LAmB following prophylaxis in liver transplant patients | Peritoneal Cmax is significantly lower than in plasma (median plasma Cmax 16.7mg/L versus median peritoneal Cmax 0.47mg/L) |

***Table S2: Table with complete list of included studies***

*LAmB=Liposomal Amphotericin B; IQR=Interquartile Range; M=Male; F=Female; NR= Not Recorded; HCT=Haematopoetic Cell Transplantation; mg=milligram; kg=kilogram; OD=Once Daily; IFD=Invasive Fungal Disease; PK=Pharmacokinetic; IMI=Invasive Mould Infection; AlloSCT=Allograft Cell Transplantation; ALL=Acute Lymphoblastic Leukaemia; BMT= Bone Marrow Transplant; LFTs= Liver Function Tests.*

**Table S3: GRADE Quality Assessment Table**

| **Paper Authors** | **Study Type** | **Initial Quality of Evidence Grade** | **Factors Reducing Quality of Evidence** | **Factors Increasing Quality of Evidence** | **Final Quality of Evidence Grade** |
| --- | --- | --- | --- | --- | --- |
| Ferreras-Antolin *et al.* 2022 ^7^ | Point Prevalence Study (Observational) | Low | -Only point prevalence data included  -No data on dosing/frequency  -No clinical or safety outcomes captured | -Large study- 275 prescriptions for prophylactic LAmB  -Recent publication  -Multi centre approach | Low |
| Mendoza-Palomar *et al.* 2021 ^8^ | Point Prevalence Study (Observational) | Low | -Only point prevalence data included  -Small-moderate sized study  - Only 55 prescriptions for prophylactic LAmB  -No data on dosing/frequency  -No clinical or safety outcomes captured | -Multi-centre  -Clear inclusion criteria | Very-Low |
| Vissing *et al.* 2021 ^25^ | Retrospective Cohort Study (Observational) | Low | -Patient characteristic not adequately described  -Follow-up duration unclear | -Moderate sized study with 62 children included  -Primary outcome prevention of invasive aspergillosis (prevention of IFD)  -Dosing strategy consistent and well described | Low |
| Döring *et al.* 2012 ^29^ | Retrospective Cohort Study (Observational) | Low | -Retrospective  -Dose altered in significant cohort of patients with suspicion of fungal infection (to 3mg/kg)  -Follow-up period only 3 weeks | -Comparison between LAmB and alternative antifungal (Caspofungin)  -Moderate cohort of 60 patients receiving prophylactic LAmB | Low |
| Bochennek *et al.* 2011 ^30^ | Prospective Observational Study & PK Study (Observational) | Low | -Variation in description of cases vs patients vs episodes of prescription  -Side effects subjectively assessed  -Use of historical controls | -Consistent dosing regimen  -Primary outcome prevention of fungal infection with standardised definitions | Low |
| Arrieta *et al.* 2010 ^18^ | RCT | High | -Primary outcome candida colonisation rather than IFD  -Relatively small number of total patients (20 received LAmB)  -Cohort VLBW neonates > outlier vs other studies included in review  - Non-blinded at point of treatment | -Fully randomised, bias minimised  -Similar demographics between groups | High |
| Roman *et al.* 2008 ^17^ | Clinical Trial (non-randomised) | High | -Bias introduced due to lack of randomisation & no controls  -Differences between patients and HCT episodes | -Consistent follow-up period (100 days post AlloSCT) | Moderate |
| Uhlenbrock *et al.* 2001 ^16^ | RCT | High | -Bias reduced by randomisation but small patient numbers: only 16 received prophylactic LAmB  -Comparison between prophylaxis vs early intervention  - Non-blinded | -Demographics similar between groups | Moderate |
| Meryk *et al.* 2020 ^22^ | Retrospective Cohort Study (Observational) | Low | -Minimal patients in cohort received prophylactic LAmB (27/198) | -Outcome consistent and appropriate  -Follow-up sufficient | Low |
| Mendoza-Palomar *et al.* 2020 ^24^ | Retrospective Cohort Study (Observational) | Low | - Retrospective observational, nil controls | -Large cohort, dosing well described, follow-up appropriate  - Clear statistical analysis | Low |
| Hand *et al.* 2014 ^19^ | Retrospective Cohort Study (Observational) | Low | -Retrospective  -Small cohort (total 19 patients) |  | Low |
| Strenger *et al.* 2014 ^23^ | PK Study | Low | -PK data (CSF transfer) in small cohort 14 patients  - Variable sampling times | -Novel outcome, not previously well characterised | Low |
| Satwani *et al.* 2009 ^20^ | Retrospective Cohort Study (Observational) | Low | - Retrospective analysis | -Moderate cohort (86 patients), outcome compared between RIC and MAC conditioning | Low |
| Kolve *et al.* 2009 ^31^ | Prospective Cohort Study (Observational) | Low | -Only 32/141 courses of LAmB given as prophylaxis and variable dosing and frequency  -Inconsistent reporting of exposure and groups | -Prospective | Very-Low |
| Allinson *et al.* 2008 ^32^ | Prospective Cohort Study (Observational) | Low | -Small cohort of patients (11), secondary prophylaxis |  | Low |
| Teisseyre *et al.* 2007 ^26^ | Retrospective Cohort Study (Observational) | Low | - Retrospective analysis | -Large cohort, liver transplant, consistent exposure, outcome adequately measured | Low |
| Mehta *et al.* 2006 ^21^ | PK Study | Low | -PK study only, small patient cohort |  | Low |
| Stuecklin-Utsch *et al.* 2002 ^33^ | Retrospective Cohort Study (Observational) | Low | - Retrosepctive analyssi  -Primary outcome not aligned with others | -Useful assessment of alternative outcomes | Low |
| Ringdén *et al.* 1997 ^27^ | Retrospective Cohort Study (Observational) | Low | -Retrospective analysis in single unit  -Exposure difficult to adequately assess given inconsistent dosing  -Cohort difficult to ascertain given episodes vs patients  -Potential confounding as patients not matched for different background diagnoses (significant differences between BMT vs liver/renal transplant) |  | Very-Low |
| Tortora *et al.* 2022 ^28^ | PK Study | Low | -PK study only, very small cohort of patients received prophylactic LAmB (3/6) |  | Very-Low |

**Table S4: Details of the Pharmacokinetic Studies included**

| **Author and Year of Publication** | **Study Type** | **Population** | **Patients Included in PK analysis** | **Dosing of LAmB** | **Outcome** | **CMax** | **AUC** | **Half-Life** |
| --- | --- | --- | --- | --- | --- | --- | --- | --- |
| Mehta *et al.* 2006 ^21^ | PK study | Paediatric HCT patients <10 years of age | 14 patients | 10mg/kg once weekly | PK profiling of serum levels of LAmB following IV administration, including trough serum concentration, AUC and total body clearance. | Cmax after first dose - mean 2.71 mg/L (range 2.1-3.4 mg/L) versus 4th dose - mean 3.02 mg/L (range 2.6-3.8 mg/L)- no significant difference. | AUC higher at week 4 (105-462 mg/h/L) than after single dose (79-275 mg/h/L), p <0.05. | Elimination half life ranged from 28.5 to 197.5 hours (median 45 hours) |
| Bochennek *et al*. 2011 ^30^ | Prospective observational cohort study also containing PK data | Paediatric haematology patients (43/44- 97.7% haematological malignancy) | Subset of 5 patients (83 patients in total study) | 2.5mg/kg twice weekly | LAmB trough and peak serum levels in random subset of 5 patients, after median 35 doses of LAmB. | Cmax median 27.5 mg/L (range 24.4-56.2mg/L); Cmin median 0.64 mg/L (range 0.22-6.19mg/L)- after median 35 doses (range, 15-66) of LAmB. | Not described | Not described |
| Strenger *et al*. 2014 ^23^ | PK study | Paediatric haematology and oncology patients (11/14- 78.6% haematological malignancy) | 14 patients | 3mg/kg on alternate days | Analysing serum vs CSF LAmB concentrations following IV administration of LAmB | CSF levels 1-100 hours post infusion ranged from 0.01-0.12 mg/L (one case 0.539 mg/L 2hours post infusion), with concurrent serum levels 1000-fold higher from 3 mg/L to 75 mg/L. | Not described. | CSF levels maintained a steady-state for longer than 48h.  Median transfer rate of 0.13% (range, 0.02-0.92%) from serum to CSF. Clear correlation between time after drug infusion and transfer rate found (r=0.801, p<0.001) with increasing time interval from drug administration and CSF puncture, the calculated transfer rate increased. |
| Tortora *et al*. 2022 ^28^ | PK study | Paediatric liver transplant patients | 6 patients | 3mg/kg once only | Analysing serum vs peritoneal LAmB concetrations (Cmin and Cmax) following IV administration of LAmB | At first TDM, median Cmax in plasma 16.71 mg/L (IQR 8.01-22.05) and peritoneal 0.47 (IQR 0.28-0.9 mg/L). Median Cmin in plasma 1.33 mg/L (IQR 0.52-5.36 mg/L) and peritoneal 0.60 mg/L (IQR 0.29-1.19 mg/L). Peritoneal Cmax was significantly lower than plasma (p<0.01) but both peritoneal Cmax and Cmin were in the therapeutic range (0.2-3.0 mg/L). | Not described | Not described |

***Table S4****:* ***Details of the Pharmacokinetic Studies included***

*LAmB=Liposomal Amphotericin B; HCT= Haematopoeitic Cell Transplantation; TDM= Therapeutic Drug Monitoring; PK= Pharmacokinetic; PK/PD= Pharmacokinetic/Pharmacodynamic; mg/L= milligram/litre; Cmax= Maximum concentration; Cmin= Minimum Concentration; IQR= interquartile range; mg/h/L= milligram/hour/litre; IV= Intravenous; AUC= Area Under Curve.*
